# Polyetheretherketone Double Functionalization with Bioactive Peptides Improves Human Osteoblast Response

**DOI:** 10.3390/biomimetics9120767

**Published:** 2024-12-17

**Authors:** Leonardo Cassari, Cristian Balducci, Grazia M. L. Messina, Giovanna Iucci, Chiara Battocchio, Federica Bertelà, Giovanni Lucchetta, Trevor Coward, Lucy Di Silvio, Giovanni Marletta, Annj Zamuner, Paola Brun, Monica Dettin

**Affiliations:** 1 Department of Industrial Engineering, University of Padova, Via Marzolo 9, 35131 Padova, Italy; leonardo.cassari@phd.unipd.it (L.C.); cristian.balducci@phd.unipd.it (C.B.); giovanni.lucchetta@unipd.it (G.L.); annj.zamuner@unipd.it (A.Z.); 2 Laboratory for Molecular Surface and Nanotechnology (LAMSUN), Department of Chemical Sciences, University of Catania and CSGI, Viale A. Doria 6, 95125 Catania, Italy; gml.messina@unict.it (G.M.L.M.); gmarletta@unict.it (G.M.); 3 Department of Science, Roma Tre University, Via della Vasca Navale 79, 00146 Roma, Italy; giovanna.iucci@uniroma3.it (G.I.); chiara.battocchio@uniroma3.it (C.B.); federica.bertela@uniroma3.it (F.B.); 4 Faculty of Dentistry, Oral & Craniofacial Sciences, King’s College London, London SE1 9RT, UK; trevor.coward@kcl.ac.uk (T.C.); lucy.di_silvio@kcl.ac.uk (L.D.S.); 5 Department of Civil, Architectural and Environmental Engineering, University of Padova, Via Marzolo 9, 35131 Padova, Italy; 6 Department of Molecular Medicine, University of Padova, Via A. Gabelli 63, 35121 Padova, Italy; paola.brun.1@unipd.it

**Keywords:** PEEK, EAK, GBMP1α, bone tissue engineering

## Abstract

In recent years, the demand for orthopedic implants has surged due to increased life expectancy, necessitating the need for materials that better mimic the biomechanical properties of human bone. Traditional metal implants, despite their mechanical superiority and biocompatibility, often face challenges such as mismatched elastic modulus and ion release, leading to complications and implant failures. Polyetheretherketone (PEEK), a semi-crystalline polymer with an aromatic backbone, presents a promising alternative due to its adjustable elastic modulus and compatibility with bone tissue. This study explores the functionalization of sandblasted 3D-printed PEEK disks with the bioactive peptides Aoa-GBMP1α and Aoa-EAK to enhance human osteoblast response. Aoa-GBMP1α reproduces 48–69 trait of Bone Morphogenetic Protein 2 (BMP-2), whereas Aoa-EAK is a self-assembling peptide mimicking extracellular matrix (ECM) fibrous structure. Superficial characterization included X-ray photoelectron spectroscopy (XPS), white light interferometer analysis, static water contact angle (S-WCA), and force spectroscopy (AFM-FS). Biological assays demonstrated a significant increase in human osteoblast (HOB) proliferation, calcium deposition, and expression of osteogenic genes (*RUNX2*, *SPP1*, and *VTN*) on functionalized PEEK compared to non-functionalized controls. The findings suggest that dual peptide-functionalized PEEK holds significant potential for advancing orthopedic implant technology.

## 1. Introduction

The rise in life expectancy across Western countries has created a growing need for orthopedic implants to address bone and cartilage disorders [1]. Historically, metals have been the preferred material for these implants due to their outstanding oxidation resistance, excellent mechanical strength, and biocompatibility [2]. However, the disparity between the elastic modulus of these metals and cortical bone, coupled with the release of metal ions from the implant surface, can result in complications and, in some cases, implant failure. Additionally, most polymeric materials cannot withstand repeated loads without undergoing permanent deformation [3].

Polyetheretherketone (PEEK) stands out as an exception to the limitations of traditional materials. PEEK is a linear, semi-crystalline polymer with an aromatic backbone that includes 1,4-disubstituted phenyl groups separated by ether (-O-) and carbonyl (-CO-) linkages. Its biomechanical properties closely resemble those of human bone, making it a promising polymeric alternative to conventional metal implants. With an elastic modulus around 3.6 GPa, PEEK can be reinforced with additives, such as carbon, to achieve a modulus comparable to that of cortical bone (approximately 18 GPa) [4,5,6]. Due to its exceptional mechanical properties, PEEK was proposed as a replacement for metal components in orthopedic implants in the late 1990s, with applications in femoral prostheses and hip joints [7]. More recently, PEEK has also gained recognition in dentistry for its favorable biomechanical and esthetic characteristics [8].

Scientific interest in PEEK has grown due to its potential in 3D printing. As a thermoplastic polymer, it can be molded within its melting temperature range [9,10], which, at around 343 °C, is relatively high compared to other polymers in its class [4,10]. PEEK demonstrates high resistance to thermal, organic, aqueous, and biodegradation, which further supports its biocompatibility, as it induces only mild inflammatory responses upon implantation [11,12].

However, the hydrophobic surface of PEEK makes it bio-inert, hindering protein adsorption and cell adhesion [13]. Enhancing cell interaction is particularly valuable in orthopedic prostheses, especially for elderly patients or those with multiple conditions, where successful osseointegration is critical. Improved osseointegration promotes faster bone growth directly in contact with the implant surface, enhancing its stability. Consequently, surface modification of PEEK to encourage cell recognition through biochemical signals, which is the aim of this work, is an exciting challenge [14].

Various physical methods for modifying the surface of PEEK have been explored, including thermal spraying [15], pulsed laser treatment [16], ion sputtering [17], sandblasting [18], electrochemical techniques [19], and plasma (oxygen) treatment [20,21].

Previous research has introduced three main approaches for PEEK functionalization. Yakufu et al. promoted cell adhesion or proliferation by anchoring signaling sequences to isocyanate or amino groups introduced by reducing PEEK’s carbonyl groups to alcohol groups [22,23,24]. Alternatively, in our previous studies, we functionalized PEEK ketones with aminooxy groups (Aoa) in peptide sequences [25,26] to achieve covalent bonding through oxime formation. This method offers a straightforward procedure that requires no additional reagents [27,28]. We also explored a more versatile approach using photoactivable bioactive peptides with an azido group incorporated into the peptide sequence. Upon photoactivation, the azido group forms a nitrene that can react with various molecules [29], providing flexibility for diverse applications [26].

In our previous study [26], we grafted the GBMP1α peptide, reproducing the sequence 48–69 from Bone Morphogenetic Protein 2 (BMP-2), onto the PEEK surface. This functionalization led to a significant increase in human osteoblasts (HOB) colonization, with homogeneous cell adherence observed and without introducing cytotoxicity within 48 h. Furthermore, the functionalization with GBMP1α significantly enhanced the proliferation rate of HOB and the expression of *SOX9, RUNX2, ALP*, and *OCN* genes. Finally, calcium deposition was also significantly increased by the GBMP1α grafting.

Starting from these promising results, in this work, to better mimic the multi-component signals of native extracellular matrix, PEEK disks were enriched simultaneously with GBMP1α and the self-assembling peptide EAK. The latter peptide produces a fibrous scaffold mimicking the extracellular matrix environment [30], enhancing cell–matrix interactions and facilitating osteoconduction and osteoinduction in vivo [31].

GBMP1α and EAK were both grafted covalently via oxime. It is well known that HOBs prefer rough surfaces [32]; consequently, different PEEK surfaces were assessed in a proliferation test, and the better one was functionalized with both bioactive peptides. In the last instance, this work investigated the potential synergistic effects of double functionalization and surface roughness.

Water contact angle and force spectroscopy analysis explored how the functionalization with the two peptides affects the surface properties of the polymer. Biological assays confirmed a better HOB response to double-functionalized PEEK vs. control.

## 2. Materials and Methods

### 2.1. Materials

Acetic acid (AcOH), acetone, acetonitrile, Fmoc-protected amino acids, Bis-Boc-aminooxy acetic acid (Aoa), dichloromethane (DCM), N,N-diisopropylethylamine (DIPEA), N,N-dimethylformamide (DMF), ethyl cyano(hydroxyimino)acetate (Oxyma Pure), methanol, Rink-Amide MBHA resin, triethylsilane (TES), and 2-(1H-benzotriazol-1-yl)-1,1,3,3-tetramethyluronium hexafluorophosphate (HBTU) were purchased from Merck Millipore (Burlington, MA, USA). Diethyl ether and trifluoroacetic acid (TFA) were obtained from Biosolve (Valkenswaard, Holland). Isopropanol for HPLC (free from aldehyde and ketone impurities) was purchased from PanReac AppliChem (Darmstadt, Germany). Ethanol was purchased from Carlo Erba (Milan, Italy), and N-Methyl-2-pyrrolidone (NMP) was acquired from Iris Biotech GmbH (Marktredwitz, Germany).

### 2.2. Peptides Preparation

Aoa-GBMP1α and Aoa-EAK peptides (Table 1) have been synthesized.

The solid-phase syntheses of the peptides were performed with Fmoc chemistry using the Rink amide MBHA resin (substitution 0.62 mmol/g) as solid support by a Syro I synthesizer (Multisyntech, Witten, Germany).

The list of amino acids used is as follows: Fmoc-Ala-OH, Fmoc-Asn(Trt), Fmoc-Asp(OtBu)-OH, Fmoc-Phe-OH, Fmoc-Glu(OtBu)-OH, Fmoc-Gln(Trt)-OH, Fmoc-His(Trt)-OH, Fmoc-Ile-OH, Fmoc-Leu-OH, Fmoc-Lys(Boc)-OH, Fmoc-Pro-OH, Fmoc-Ser(tBu)-OH, Fmoc-Thr(tBu)-OH, Fmoc-Val-OH, Fmoc-7-amino-heptanoic acid-OH, and Bis(Boc)-Aoa-OH. The spacer increases the flexibility of the peptide and separates the bioactive sequences from the PEEK surface.

Each amino acid was incorporated using a double coupling process with 5 equivalents (eq.) of the amino acid, 5 eq. of the activating agent HBTU/Oxyma Pure, and 10 eq. of DIPEA relative to the reactive groups on the resin for 45 min at room temperature (RT). Aoa was introduced into the sequence by a similar double coupling method, using 5 eq. of Bis(Boc)-Aoa-OH, 5 eq. of the activating agent HATU/Oxyma Pure, and 10 eq. of collidine with respect to the resin’s reactive groups, also for 45 min at RT.

Upon completing chain elongation, the peptides were cleaved from the resin and deprotected from Bis-Boc and side-chain protecting groups by treating with 4.75 mL TFA, 0.125 mL TES, and 0.125 mL H_2_O for 1.5 h at room temperature with magnetic stirring. The resin was filtered off, and the solution was concentrated and added with cold diethyl ether to precipitate the product. The crude peptide was finally filtered and dried. Finally, each peptide was purified by reverse-phase high-performance liquid chromatography (RP-HPLC) using a Vydac C18 column (5 μm, 300 Å, 4.6 × 250 mm). Conditions: 27 to 37% B in 20 min for Aoa-GBMP1α and 25 to 35% B in 15 min for Aoa-EAK. The degrees of purity of the obtained peptide are over 94% for Aoa-GBMP1α and over 90% for Aoa-EAK. The identity of the peptide was determined by mass spectrometry. Aoa-EAK was analyzed with ESI-TOF (Mariner System 5220, Applied Biosystem, PerkinElmer, Foster City, CA, USA). The theoretical mass of this peptide is 1813.99 Da, and the experimental mass was 1813.66 Da. Aoa-GBMP1α was analyzed with MALDI-TOF (SCIEX-TOF 4800 instrument, Foster City, CA, USA). The theoretical mass of this peptide is 2587.86 Da, and the experimental mass was 2587.81 Da.

### 2.3. PEEK Sample Preparation

**Figure 1:**
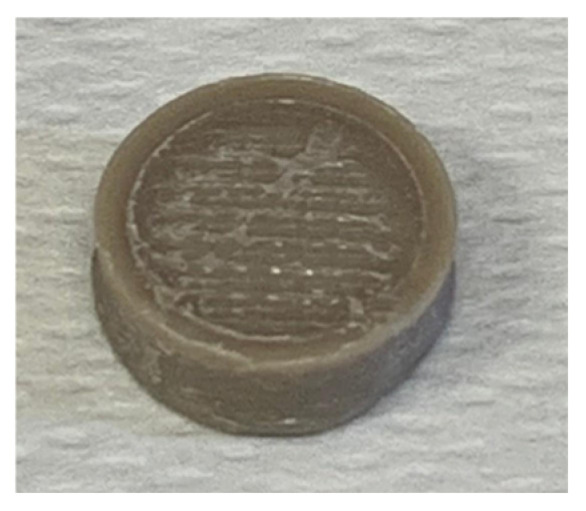
3D-printed PEEK disk. Diameter 1 cm, height 0.4 cm.

The PEEK sample preparation process is the same as already described in our previous papers [25,26]: it involved using Autodesk Fusion 360, a computer-aided design (CAD) software (Version number 16985.2.0.0) from San Rafael, CA, USA, to create PEEK disk files. These files were exported in STL format and imported into Simplify 3D software (Version 5.0, Cincinnati, OH, USA). In Simplify 3D, the files were sliced to generate G-code suitable for 3D printing. Printing was performed on an Apium P155 PEEK filament printer, manufactured by Apium Additive Technologies GmbH in Willy, Karlsruhe, Germany, using 1.75 mm PEEK filament from Vitrex Rotherham (Rotherham, UK). The filament was extruded through a 0.4 mm diameter nozzle at 485 °C onto a print bed held at 130 °C. The print speed was set to 33.3 mm/s. The resulting 3D-printed PEEK disks had a flat surface on one side and a patterned surface on the other, with dimensions of 10 mm in diameter, 4 mm in height, and 100% infill. The patterned upper surface was achieved by extruding the PEEK filament in both horizontal and vertical lines, with a 0.5 mm step across the surface of the disk. The result is shown in Figure 1.

**Figure 2:**
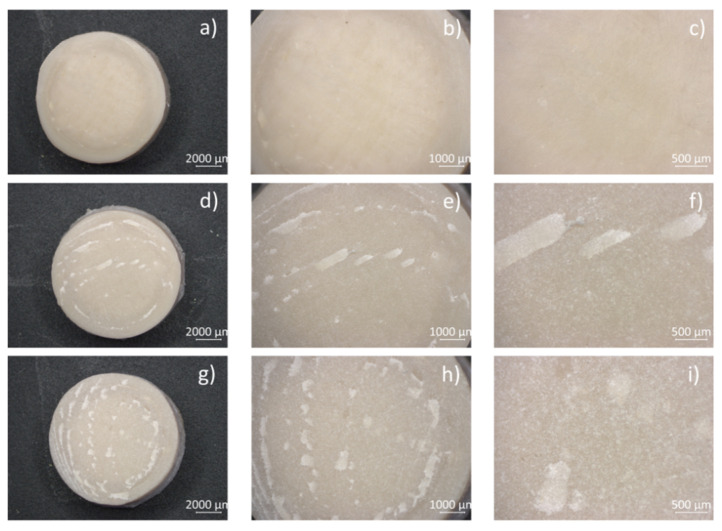
Microscope images of 2000 grit paper smooth PEEK (**a**–**c**), R60-PEEK (**d**–**f**), and R110-PEEK (**g**–**i**). Magnification at 6.3× (**a**,**d**,**g**), 12.5× (**b**,**e**,**h**), and 32× (**c**,**f**,**i**).

The disks have been smoothed with a 2000 grit paper, from now on just called PEEK (Figure 2a–c). Therefore, a dental sandblaster (Emmevi Apparecchi Dentali, Settimo Milanese, Italy) has been used to modify the surface roughness of smooth PEEK disks. The PEEK disks were kept at 2–3 cm from the compressed air outlet, which was kept constant by means of a manual controller. Each disk was held in the blasting machine for approximately 10 s. Two corundom sizes were used to define different superficial roughness: 60 μm to obtain R60-PEEK (Figure 2d–f) and 110 μm to obtain R110-PEEK (Figure 2g–i).

### 2.4. PEEK Surface Functionalization

To functionalize PEEK samples with both Aoa-GBMP1α and Aoa-EAK simultaneously, we employed covalent oxime-based functionalization [25,26]. This method establishes a chemoselective ligation between the amino-oxy groups at the N-terminus of the peptide chains and the ketonic groups on the PEEK surface. Unlike the Schiff base anchoring method previously proposed by Becker et al. [33] for PEEK functionalization, oxime anchoring does not require reduction.

Differently from our previous works [25,26], for the functionalization with both Aoa-GBMP1α and Aoa-EAK via oxime, the solvent role was investigated for a further optimization of the functionalizing protocol. Due to the hydrophobic nature of PEEK, several organic solvents have been studied to improve the interactions between the solvent and the polymer surface. A necessary feature of the solvent was to be free from any contaminants such as aldehydes or ketones that may react to the Aoa group of the peptide. The solvents, 50% DMF/MilliQ water, 50% dimethyl sulfoxide (DMSO)/MilliQ water, and DMSO, have been chosen since they had been already employed in functionalization via oxime [34,35]. To select the best solvent, 2 μL of each solution (plus MilliQ water as a control) have been dropped on the surface of PEEK. Firstly, the choice of solvent was made qualitatively by assessing the best spreading, and based on this test, DMSO was selected as the best solvent, as shown in Section 3.2.1.

The incidence of solvent on functionalization yield was then assessed in a preliminary XPS experiment using only Aoa-EAK peptide. 10^−4^ M Aoa-EAK in 1 eq of AcOH in DMSO [35,36] has been then leaved on PEEK disks for 1 h at RT. Based on the results of the XPS analysis shown in Section 3.2.2, the previous choice has been confirmed, and the functionalization with two peptides simultaneously has been conducted in the organic solvent.

**Figure 3:**
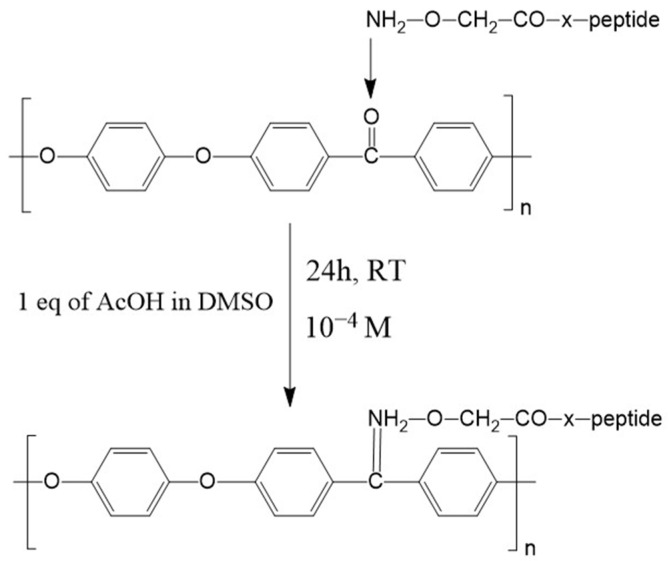
PEEK functionalization via oxime formation scheme.

The two peptides were dissolved in 1 eq of AcOH in DMSO at a concentration of 10^−4^ M each, and the reaction (Figure 3) took place at RT for 24 h. After the end of the reaction time, the disks were subjected to three rinses with DMSO and three rinses with MilliQ water.

### 2.5. Superficial Characterization Assays

#### 2.5.1. X-Ray Photoelectron Spectroscopy

XPS investigations were carried out in a home-made instrument, consisting of a preparation and an analysis chamber, that was previously described in [37]. Samples underwent overnight outgassing in the preparation chamber with a base pressure of approximately 10^−8^ Torr and were subsequently moved to the analysis chamber, where the vacuum pressure ranged between 10^−8^ and 10^−10^ Torr during measurements. XPS analysis utilized non-monochromatized Mg Kα X-ray radiation (1253.6 eV). Energy calibration for all spectra was based on the C1s signal of aromatic-aliphatic carbons, set at a binding energy (BE) of 284.7 eV. After a Shirley-type background subtraction, Gaussian functions were used for curve-fitting of the C1s, N1s, and O1s spectra, with a uniform full width at half maximum (FWHM) value applied to all photoemission peaks within each spectrum. Atomic ratios and surface atomic concentrations were calculated from peak areas using Scofield’s atomic sensitivity factors for accurate quantification.

#### 2.5.2. Non-Contact Profilometry

The mapping of the surface topography of PEEK, R60-PEEK, and R110-PEEK scaffolds was carried out with a 3D confocal microscope, Sensofar Neox (Sensofar Tech, Terrassa, Spain), at 20× magnification. Height maps of area 4.41 × 3.86 mm were extracted and leveled by subtracting the mean plan. Surface topography measurements were conducted according to ISO 25178-2:2021 [38]. Key parameters, including Spk (peak height), Svk (valley depth), Sa (arithmetic mean height), Sku (kurtosis), Smean (root mean square height), Sp (maximum height of the surface), Sq (root mean square of height), Ssk (skewness), Sv (maximum pit or peak volume), and Sz (maximum height of the surface above the material ratio curve), were quantified to characterize the surface features of the scaffolds.

#### 2.5.3. Water Contact Angle

Surface wettability was assessed by measuring the static water contact angle (WCA). An OCA30 instrument from Dataphysics was employed at a temperature of 25 °C and a relative humidity of 65%. Droplets of probe liquid with a volume of 2 μL were carefully dispensed onto various regions of each sample surface. Digital image analysis was used to determine the static contact angle, which was obtained as the tangent to the drop at the point where it contacted the surface, both on the right and left sides. At least three measurements were taken for each sample, and the results were averaged.

#### 2.5.4. Atomic Force Spectroscopy

Atomic Force Microscopy (AFM) analysis was performed on functionalized PEEK disks at room temperature to evaluate the influence of peptides on the mechanical properties of the interfaces. The Young’s modulus was calculated from force-distance curves acquired with an NTEGRA AFM (NT-MDT, Moscow, Russia). Stiff single-crystal silicon cantilevers with a symmetric tip shape (model Tap300Al-G, BudgetSensors, Bulgaria; nominal frequency: 300 kHz, nominal spring constant: 40 Nm^−1^, tip radius < 10 nm) were employed. The Sader method was utilized to determine the cantilever spring constant for each probe. Calibration involved performing a force curve on a hard-cleaned substrate (<100> silicon wafer), where no indentation occurred. The calibration was necessary to ascertain the sensitivity and spring constant of the probes. Young’s modulus was calculated from experimental force-distance curves using the Derjaguin-Müller-Toporov (DMT) model, represented by the following equation:

(1) $$F+F_{ad}=\frac{4E_{s}}{3\left(1-\vartheta_{s}^{2}\right)}R^{\frac{1}{2}}\delta^{\frac{3}{2}}$$

where F represents the applied force, F_ad_ is the adhesion force, E_s_ is Young’s modulus, ϑ_s_ is the Poisson’s ratio of the sample, R is the radius of the spherical indenter, and δ is the elastic indentation depth. Each surface was indented in multiple areas, collecting approximately 300 force-distance curves. The approach rate of the tip to the sample for all force curves was set at 0.3 µm/s. Young’s modulus was determined by fitting the experimental curves in the elastic region using the DMT model, and the distribution was analyzed with OriginPro 8.5 software.

For adhesion force measurements, Au-coated silicon nitride tips with spring constants ranging from 0.003 to 0.13 N/m were used. Individual spring constants were calibrated using the Sader method. The adhesion force was defined as the pull-off force required to separate the AFM tip from the surface. Approximately 300 approach–retract cycles were collected for each system, with a tip-sample approach speed of 0.3 µm/s. Adhesion force was calculated from the deflection distance of the cantilever and the cantilever spring constant using Hooke’s law:

F = k × ΔL,(2)

where F represents the force (nN), k is the spring constant of the cantilever, and ΔL denotes the deflection distance (nm).

### 2.6. Biologic Assays

#### 2.6.1. Cell Culture

Primary HOB cells were obtained from residual cortical mandible bone collected during the extraction of deeply impacted third molars from a 39-year-old male (research protocol No. 4899/AO/20, Ethics Committee Azienda Ospedaliera/University of Padua). Initially, bone fragments were cultured in DMEM/F12 (1:1) supplemented with 20% *v/v* fetal bovine serum, 1% *v/v* sodium pyruvate, 1% *v/v* nonessential amino acids, 1% *v/v* antibiotic–antimycotic solution, and 1 U/mL insulin (all from Gibco, Invitrogen, Milan, Italy) until cells began migrating from the tissue. Once confluent, the bone fragments were removed with sterile tweezers. Cells were then detached using trypsin–EDTA (Gibco) and cultured either in complete culture medium (CM) or differentiation culture medium (DM), with DM defined as CM supplemented with 50 μg/mL ascorbic acid, 10 nM dexamethasone, and 10 mM β-glycerophosphate (all from Sigma, Milan, Italy). A total of 2 × 10^5^ cells were seeded onto each sample.

#### 2.6.2. Proliferation Assay

Proliferation tests were first conducted on (i) non-functionalized PEEK disks with varying surface roughness to determine which surface condition enhances HOB proliferation and differentiation and then (ii) on PEEK samples functionalized with both peptides simultaneously. The details are presented as follows:

(i) Cells were incubated at 37 °C for 10 min in prewarmed PBS containing 0.1% *v/v* BSA and 25 mM carboxyfluorescein diacetate succinimidyl ester (CFSE, Molecular Probe, Invitrogen). To quench the staining process, five volumes of ice-cold culture medium were added. The cells were then centrifuged (1600 rpm, 6 min) and counted using Trypan blue. Cells were suspended in fresh culture medium, seeded onto each sample, and cultured at 37 °C for 7 days. Cell proliferation was measured by examining the distribution of the fluorescent dye between daughter cells in 50,000 events using a BD FACS-Calibur flow cytometer. Data are reported as the percentage of fluorescent positive cells.
(ii) The proliferation of osteoblasts on PEEK and PEEK disks functionalized with the two peptides was evaluated using the AlamarBlue^TM^ assay (alamarBlue Cell Viability Reagent, Invitrogen, ThermoFisher Scientific, Waltham, MA, USA). Fluorescence measurements were taken at a test wavelength of 570 nm and a reference wavelength of 630 nm using a microplate reader (Opsys MRTM 96-well microplate reader, Dynex Technologies, Chantilly, VA, USA). To ensure the accuracy of the results, each sample was measured in triplicate. Data were collected at two time points: 1 and 7 days from cell seeding.

#### 2.6.3. Mineralization Assay

Cells were cultured onto each sample for 7 days (an incubation time previously set in our experiments as the most effective for calcium deposition [39]) and then assessed for mineralization. Cell monolayers were washed with distilled water, and samples were stained with 40 mM Alizarin Red (pH 4.2) for 40 min in darkness at room temperature. The samples were then incubated at 85 °C for 10 min, followed by centrifugation (10 min at 13,000 rpm). The pH of the supernatants was neutralized before measuring the absorbance of Alizarin Red at 405 nm using a MultiPlateReader VictorX2 (PerkinElmer Inc., Waltham, MA, USA) microplate reader.

#### 2.6.4. Gene Expression Assay

The quantification of genes encoding three fundamental protein sequences for osteogenesis was performed: secreted phosphoprotein 1 (*SPP1*), Runt-related transcription factor 2 (*RUNX2*), and Vitronectin (*VTN*), which are encoded by the homonymous genes. *RUNX2* is recognized for its role in regulating the proliferation of osteoblast progenitors and their differentiation into mature osteoblasts [40]. *SPP1* is a protein crucial for osteoblast adhesion and bone homeostasis, also found in the early stage of bone healing, which can also support the osteogenic differentiation of osteoprogenitor cells [41,42]. *VTN* mediates cell attachment, spreading, and migration to the ECM [43]. Furthermore, the adhesion of MSC to *VTN* promotes osteogenic differentiation by increasing *OCN*, *COL1*, and *ALP* levels [44]. It also induces mineralization in the presence or absence of osteogenic-inducing supplements.

Primary HOB cells (2 × 10^5^) were cultured in direct contact with PEEK samples for 24 h in 1 mL of either CM or DM. Following this, specific mRNA transcript levels for *SPP1*, *RUNX2*, and *VTN* were measured. Total RNA was extracted from cells attached to the biomaterials using the SV Total RNA Isolation System kit (Promega, Milan, Italy), and any contaminating DNA was removed by DNase I digestion. For cDNA synthesis, 5 µg of total RNA were reverse-transcribed using Moloney murine leukemia virus reverse transcriptase (Applied Biosystems, Milan, Italy) with random primers. Quantitative polymerase chain reaction (qPCR) was performed on an ABI Prism 7700 sequence detector (Applied Biosystems) for 40 cycles at an annealing temperature of 60 °C. The reactions were carried out with TaqMan Universal PCR Master Mix and Universal Probe Library (Roche, Milan, Italy), which included a fluorescent dye, as per the manufacturer’s instructions. Expression levels of target genes were normalized to the endogenous control gene glyceraldehyde-3-phosphate dehydrogenase (GAPDH). Data were further normalized using the 2^−∆∆Ct^ method and are presented as fold change relative to osteoblasts cultured on cell-treated plastic. The oligonucleotide sequences and probes are provided in Table 2.

#### 2.6.5. Statistical Analysis

All experiments were conducted in triplicate, with results presented as mean values accompanied by standard deviations. Statistical analysis and data manipulation were performed using Prism software (GraphPad Software 9). The outcomes of the Alizarin Red S and qPCR assays were analyzed using a one-way analysis of variance (ANOVA), followed by Tukey’s multiple comparisons test for detailed condition comparisons. The proliferation data were analyzed using two-way ANOVA, followed by Tukey’s post hoc test for multiple comparisons. A significance level of 5% was used to determine the statistical significance of the results throughout this study.

## 3. Results and Discussion

### 3.1. Evaluation of Surface Roughness

PEEK disks were smoothed and then sandblasted with two different grain sizes to obtain different roughness surfaces. These samples were characterized and seeded with HOB to select the roughness that optimizes cellular response.

#### 3.1.1. Non-Contact Profilometry

The topographic data for the differently roughened surfaces of PEEK samples are presented in Table 3. The PEEK scaffold smoothed with 2000 grit paper exhibited a relatively smoother surface, with Spk and Svk values of 0.40 μm and 0.80 μm, respectively. In contrast, sandblasting with a 110 μm grain size (R110-PEEK) significantly increased the peak height (Spk = 4.25 μm) and valley depth (Svk = 4.25 μm). The intermediate profile of R60-PEEK, modified with a 60 μm grain size, showed values between the unmodified and extensively modified surfaces. The height parameters, including Sa, Sku, Smean, Sp, Sq, Ssk, Sv, and Sz, further highlight the subtle topographical changes introduced by the modification process. The profilometer microimages of all samples are reported in Supplementary Materials (Figures S1–S3).

#### 3.1.2. Water Contact Angle

WCA measurements were conducted to investigate the impact of surface roughness on surface wettability. The size of the sandblasting particles significantly influences wettability. The average values and standard deviations of contact angle are reported in Table 4. Notably, the WCA values increase with greater surface roughness. Both R60-PEEK and R110-PEEK exhibited increased hydrophobicity compared to untreated PEEK.

#### 3.1.3. Proliferation Assay on Samples of Different Roughness

**Figure 4:**
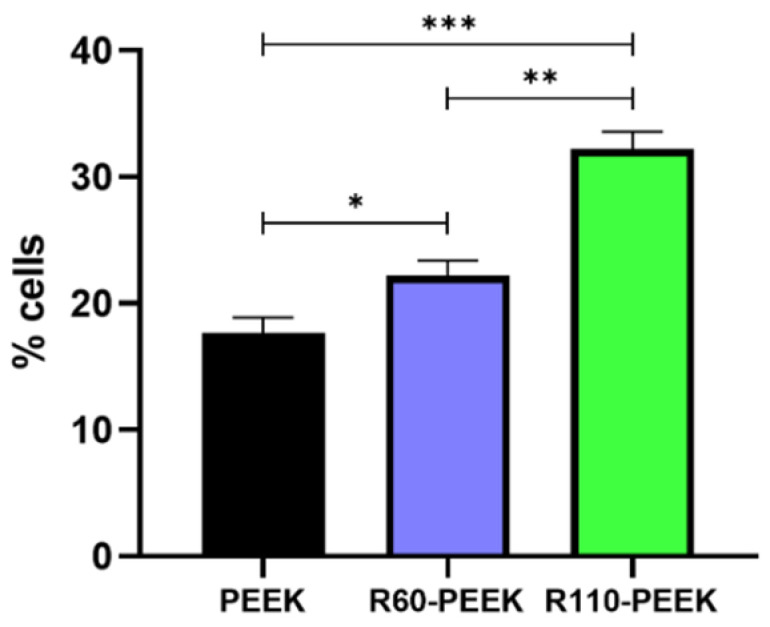
HOB proliferation test at 7 days after cell seeding. Data are reported as a percentage of CFSE-positive cells. PEEK refers to smooth samples; R60-PEEK and R110-PEEK are samples of PEEK sandblasted with a 60 μm and 110 μm grain size, respectively. Significance levels: * *p*-value < 0.05, ** *p*-value < 0.01, *** *p*-value < 0.0001.

The HOB proliferation test was performed after 7 days of cell seeding on PEEK, R60-PEEK, and R110-PEEK. This evaluation aimed to determine how HOB responded to various roughness we introduced to PEEK disks. The test revealed that both the sandblasted samples significantly improved the HOB activity compared to smooth PEEK (Figure 4). From the data, a direct correlation appeared between the level of roughness and the amount of cell proliferation. Indeed, not only did R110-PEEK have an 82% increase compared to PEEK, but also its improvement in cell activity was significant with respect to R60-PEEK (+25.7% compared to PEEK) by 45.2%.

According to the results of these assays, we decided to evaluate the combined effects of Aoa-GBMP1α and Aoa-EAK functionalization on PEEK sandblasted with 110 μm sized grains.

### 3.2. Selection of the Best Solvent for Functionalization

To increase the oxime reaction yield, different solvents [45] showing good PEEK wettability were evaluated.

#### 3.2.1. Contact Angle Evaluation for the Optimization of Functionalization Yield

**Figure 5:**
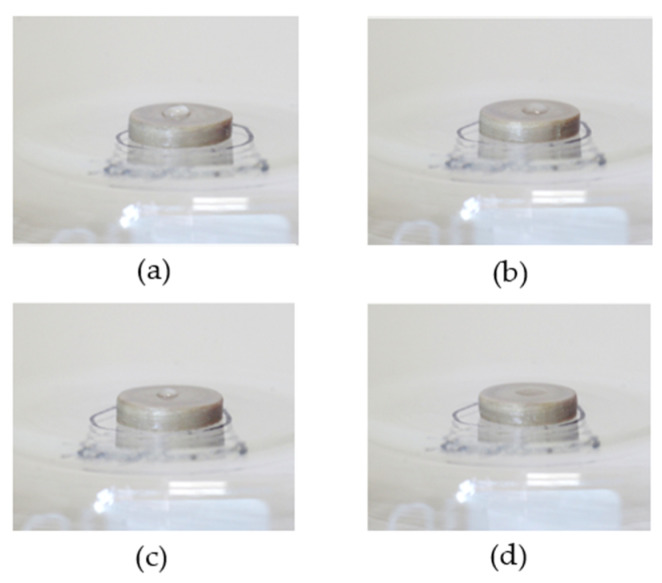
Qualitative evaluation of drop dispersion on PEEK disks of (**a**) water, (**b**) 50% DMF/MilliQ water, (**c**) 50% DMSO/MilliQ water, and (**d**) DMSO.

The assessment of PEEK wettability was carried out with 2 μL of 50% DMF/MilliQ water, 50% dimethyl sulfoxide (DMSO)/MilliQ water, DMSO, and MilliQ water as control. Firstly, the choice of solvent was made qualitatively assessing the best drop spreading. As shown in Figure 5, we noted that the organic solvents decreased the contact angle of the solvent on the surface with respect to water. We obtained the highest spreading with pure DMSO over the 50% DMF/water and 50% DMSO/water.

#### 3.2.2. XPS Analysis

**Figure 6:**
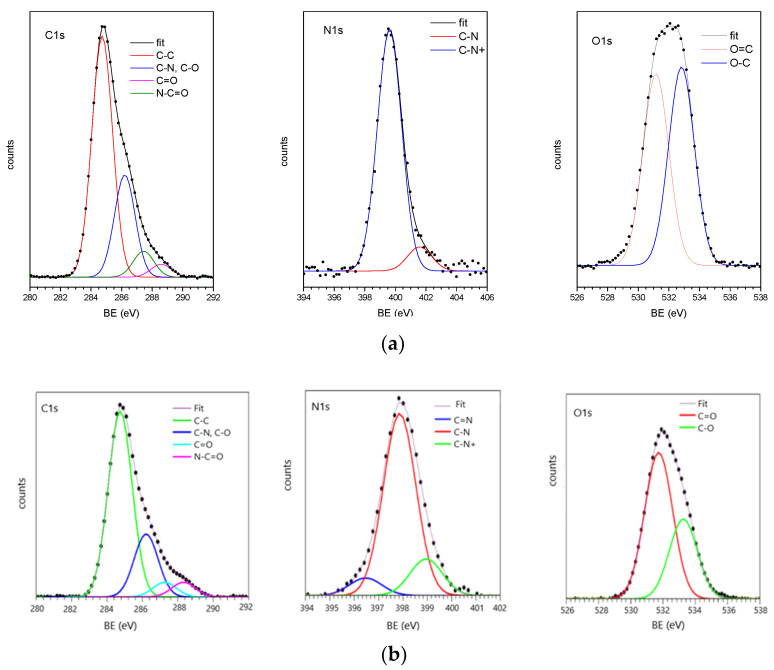
C1s, N1s, and O1s XPS spectra and relative curve-fitting analysis of sample PEEK + Aoa-EAK (**a**) in 1 eq of AcOH in DMSO and (**b**) in 40 mM monobasic sodium phosphate at pH 6; markers represent experimental points, lines fitting components, and calculated spectra.

The influence of solvent on functionalization yield was assessed by performing the reaction with DMSO or PBS; this last condition was carried out in a previously published study [26]. XPS analyses were carried out on functionalized PEEK samples. In detail, 10^−4^ M Aoa-EAK in either 1 eq of AcOH in DMSO or in 40 mM monobasic sodium phosphate at pH 6 has been left on PEEK disks for 1 h at RT. The C1s, N1s, and O1s core levels. XPS data (BE, FWHM, atomic ratios) are reported in Table S1. The C1s, N1s, and O1s spectra of the samples are shown in Figure 6.

As already discussed for similar samples [26], the C1s spectra consist of four components. The first component, fixed at 284 eV, is due to aromatic and aliphatic C-C carbons; the second at about 286.2 eV, to C-N and C-O of PEEK and of the immobilized peptide; the third at about 287.3 eV is due to the C=O carbons of PEEK; finally, the fourth component at about 288.3–288.5 eV is due to N-C=O carbons of the immobilized peptides [46]. Peptide immobilization on the PEEK surface is proved by the presence of the N1s signals typical of the peptide; N1s spectra show two components, the first one around 400 eV related to C-N nitrogens of the peptide backbone, and the second at about 402 eV to protonated nitrogens; in the spectra of PEEK + Aoa-EAK prepared in 40 mM monobasic sodium phosphate at pH 6, a low-intensity peak located at 398.5 eV and assigned to C=N carbons is also detected [47]. Finally, O1s spectra show two components, the first one, at nearly 531.5 eV, associated with C=O oxygens, the second, at 533.2 eV, with C-O oxygens.

XPS data show that both functionalization pathways were effective in the covalent attachment of the EAK peptide to the PEEK surface, as evidenced by the measured C/N ratios shown in Table 5. A slightly higher N/C ratio, related to a higher peptide content, was evidenced for the PEEK–Aoa-EAK-functionalized samples when the anchoring reaction occurred in DMSO compared to 40 mM monobasic sodium phosphate (Table 5).

### 3.3. R110-PEEK Functionalized with Aoa-GBMP1α and Aoa-EAK

#### 3.3.1. Water Contact Angle

**Figure 7:**
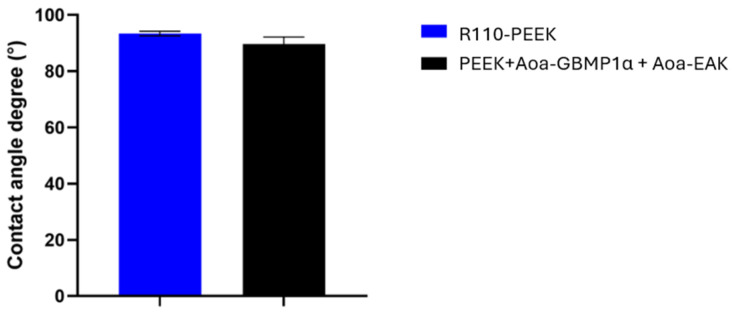
WCA results on R110-PEEK and R110-PEEK enriched with Aoa-GBMP1α and Aoa-EAK simultaneously.

The presence of Aoa-GBMP1α and Aoa-EAK on the PEEK surface did not substantially modify the surface WCA (Figure 7).

#### 3.3.2. Force Spectroscopy

**Figure 8:**
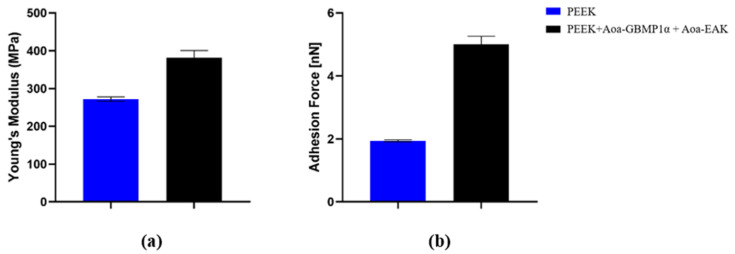
(**a**) Young’s Modulus and (**b**) force adhesion of untreated smooth PEEK and R110-PEEK enriched with Aoa-GBMP1α and Aoa-EAK simultaneously.

Force spectroscopy spotted the presence of the peptides on the polymer as a higher interchain interaction that caused a 40% increase in superficial stiffness (Figure 8a) in accordance with literature [48]. Furthermore, the force adhesion between the AFM tip and sample surfaces has grown up after the PEEK enrichment with peptides of a 2.56 factor (Figure 8b). The increased Young’s modulus may result from the peptides filling gaps between surface polymer chains, while the enhanced adhesive force likely stems from intermolecular interactions with the tip.

#### 3.3.3. Proliferation Assay

**Figure 9:**
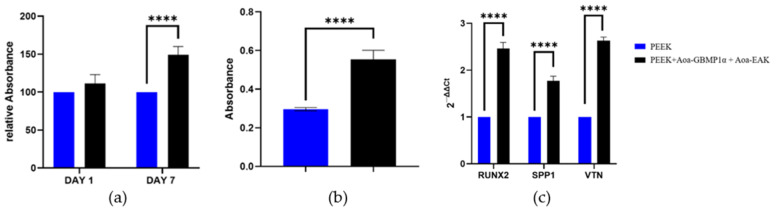
(**a**) AlamarBlueTM data obtained at days 1 and 7 following HOB seeding, (**b**) Alizarin Red assay for evaluating calcium deposition after 7 days, and (**c**) qRT-PCR analysis performed to evaluate the expression of *RUNX2*, *SPP1*, and *VTN* genes 1 day post-seeding with HOB on smooth PEEK and R110-PEEK + Aoa-GBMP1α + Aoa-EAK samples. Statistical significance: **** *p*-value < 0.0001.

The proliferation assay results for PEEK functionalized with Aoa-GBMP1α and Aoa-EAK simultaneously were recorded at 1 and 7 days after HOB seeding. At day 1, HOB proliferation on PEEK + Aoa-GBMP1α + Aoa-EAK increased by 11.5% compared to non-functionalized PEEK, though this difference was not statistically significant. However, by day 7, HOB proliferation on PEEK + Aoa-GBMP1α + Aoa-EAK showed a statistically significant increase of 49.3% compared to untreated PEEK (Figure 9a).

#### 3.3.4. Mineralization Assay

After 7 days of HOB seeding, the functionalized PEEK stimulated cells to deposit a greater amount of calcium at the extracellular compartment compared to untreated PEEK. The values of absorbance read on PEEK + Aoa-GBMP1α + Aoa-EAK were higher by 87% than PEEK (Figure 9b).

#### 3.3.5. Gene Expression Assay

qRT-PCR was employed to analyze the expression of the RUNX2, SPP1, and VTN genes in HOB cells 1 day after they had been seeded on PEEK and PEEK + Aoa-GBMP1α + Aoa-EAK. Together, the sandblasting and the biochemical enrichment statistically increased the expression of all the considered genes. After one day, RUNX2, SPP1, and VTN were upregulated by the functionalized samples compared to PEEK by 146.5%, 77.0%, and 163.0%, respectively (Figure 9c).

## 4. Conclusions

In our previous works, the advantage of PEEK covalent functionalization with AoaGBMP1α was demonstrated [26]. In this paper, HOB response was guided by a double peptide functionalization on a sandblasted PEEK surface. Surface roughness was found to promote cell growth compared to smooth PEEK surfaces as expected [32]. The covalent attachment of peptides through oxime linkages was optimized by changing the solvent of functionalization. XPS indicated that the samples functionalized in DMSO contained higher nitrogen levels (indicating more peptide) compared to those where anchoring occurred in 40 mM PBS. The anchoring of both peptides did not modify substantially the wettability of PEEK. Force spectroscopy (FS) showed that functionalization enhanced both the Young’s modulus and adhesive force of the PEEK surface. Considering biological data, the surfaces functionalized with both peptides exhibited an increase in cell proliferation compared to the control, with a notable 49.3% increase at 7 days. Both mineralization assays and gene expression analyses for *RUNX2*, *SPP1*, and *VTN* highlighted a significant enhancement on the functionalized surfaces compared to PEEK. In conclusion, our data demonstrated that the contemporary optimization of surface roughness and bioactive peptide grafting are desirable to guide h-osteoblast response. The proposal of multiple peptides anchoring meets the need to operate more biochemical stimuli at the same time to better mimic the complexity of bone extracellular matrix.

## Figures and Tables

**Table 1:** Amino acid sequences of Aoa-GBMP1α and Aoa-EAK peptides. Aoa = Amino-oxyacetic acid; x = 7-aminoheptanoic acid. Peptides present amino groups at their N-terminus and amide groups at their C-terminus.

Name of the Peptide	Sequence
Aoa-GBMP1α	NH_2_-O-CH_2_-CO-x-Pro-Phe-Pro-Leu-Ala-Asp-His-Leu-Asn-Ser-Thr-Asn-His-Ala-Ile-Val-Gln-Thr-Leu-Val-Asn-Ser-NH_2_
Aoa-EAK	NH_2_-O-CH_2_-CO-x-Ala-Glu-Ala-Glu-Ala-Lys-Ala-Lys-Ala-Glu-Ala-Glu-Ala-Lys-Ala-Lys-NH_2_

**Table 2:** Oligonucleotide sequences utilized for qRT-PCR analysis.

Gene	Sequence
*GAPDH*	fw: 5′-acagttgccatgtagacc-3′ rv: 5′-ttgagcacagggtacttta-3′
*RUNX2*	fw: 5′-cccagtatgagagtaggtgtcc-3′ rv: 5′-gggtaagactggtcataggacc-3′
*VTN*	fw: 5′-ggaggacatcttcgagcttct-3′ rv: 5′-gctaatgaactggggctgtc-3′
*SPP1*	fw: 5′-cgcagacctgacatccagta -3′ rv: 5′-ggctgtcccaatcagaagg-3′

**Table 3:** Surface texture parameters from the 3D confocal microscopy. The samples evaluated were smooth PEEK, PEEK sandblasted with 60 μm R60-PEEK), and 110 μm (R110-PEEK) sized grain.

	PEEK	R60-PEEK	R110-PEEK
ISO 25178/Functional [38]			
Spk (μm)	0.40	1.80	4.25
Svk (μm)	0.80	1.92	4.25
ISO 25178/Height [38]			
Sa (μm)	0.27	1.39	2.34
Sku (μm)	76.56	4.53	20.83
Smean (μm)	−0.0021	0.0047	0.0743
Sp (μm)	27.25	29.08	52.02
Sq (μm)	0.44	1.79	3.33
Ssk (μm)	−0.86	0.09	1.12
Sv (μm)	12.78	13.70	26.72
Sz (μm)	40.03	42.78	78.73

**Table 4:** WCA analysis conducted on smooth and sandblasted PEEK surfaces. Reported values represent the mean ± standard deviation.

Sample	Contact Angle ± Standard Deviation
PEEK	71.35 ± 8.14
R60-PEEK	79.43 ± 6.99
R110-PEEK	87.79 ± 13.67

**Table 5:** Measurement via XPS of the N/C ratio of unmodified PEEK, PEEK + Aoa-EAK in 40 mM monobasic sodium phosphate at pH 6, and PEEK + Aoa-EAK in 1eq of AcOH in DMSO.

Sample	N/C
PEEK Control	/
PEEK + Aoa-EAK (in 40 mM monobasic sodium phosphate at pH 6)	0.056
PEEK + Aoa-EAK (in 1 eq of AcOH in dimethyl sulfoxide)	0.070

## Data Availability

The raw data supporting the conclusions of this article will be made available by the authors on request.

## Supplementary Materials

The following supporting information can be downloaded at: https://www.mdpi.com/article/10.3390/biomimetics9120767/s1, Figure S1: Profilometer microimages obtained on smooth PEEK; Figure S2: Profilometer microimages obtained on R60-PEEK; Figure S3: Profilometer microimages obtained on R110-PEEK; Table S1: XPS data (BE, FWHM, atomic ratios) of PEEK, PEEK + Aoa-EAK in 40 mM monobasic sodium phosphate at pH 6, and PEEK + Aoa-EAK in 1 eq of AcOH in DMSO samples.

## References

[1] Victor S.P., Muthu J. (2014). Polymer Ceramic Composite Materials for Orthopedic Applications—Relevance and Need for Mechanical Match and Bone Regeneration. J. Mechatron..

[2] Verma R.P. (2020). Titanium Based Biomaterial for Bone Implants: A Mini Review. Mater. Today Proc..

[3] Uebersax L., Merkle H.P., Meinel L. (2009). Biopolymer-Based Growth Factor Delivery for Tissue Repair: From Natural Concepts to Engineered Systems. Tissue Eng. Part B Rev..

[4] Williams D.F., McNamara A., Turner R.M. (1987). Potential of Polyetheretherketone (PEEK) and Carbon-Fibre-Reinforced PEEK in Medical Applications. J. Mater. Sci. Lett..

[5] Schwitalla A.D., Abou-Emara M., Spintig T., Lackmann J., Müller W.D. (2015). Finite Element Analysis of the Biomechanical Effects of PEEK Dental Implants on the Peri-Implant Bone. J. Biomech..

[6] Sarasua J.R., Remiro P.M., Pouyet J. (1996). Effects of Thermal History on Mechanical Behavior of PEEK and Its Short-Fiber Composites. Polym. Compos..

[7] Williams D. (2008). Polyetheretherketone for Long-Term Implantable Devices. Med. Device Technol..

[8] Papathanasiou I., Kamposiora P., Papavasiliou G., Ferrari M. (2020). The Use of PEEK in Digital Prosthodontics: A Narrative Review. BMC Oral Health.

[9] Honigmann P., Sharma N., Okolo B., Popp U., Msallem B., Thieringer F.M. (2018). Patient-Specific Surgical Implants Made of 3D Printed PEEK: Material, Technology, and Scope of Surgical Application. BioMed Res. Int..

[10] Basgul C., Yu T., MacDonald D.W., Siskey R., Marcolongo M., Kurtz S.M. (2018). Structure–Property Relationships for 3D-Printed PEEK Intervertebral Lumbar Cages Produced Using Fused Filament Fabrication. J. Mater. Res..

[11] Blanch-Martínez N., Arias-Herrera S., Martínez-González A. (2021). Behavior of Polyether-Ether-Ketone (PEEK) in Prostheses on Dental Implants. A Review. J. Clin. Exp. Dent..

[12] Harting R., Barth M., Bührke T., Pfefferle R.S., Petersen S. (2017). Functionalization of Polyethetherketone for Application in Dentistry and Orthopedics. BioNanoMaterials.

[13] Panayotov I.V., Orti V., Cuisinier F., Yachouh J. (2016). Polyetheretherketone (PEEK) for Medical Applications. J. Mater. Sci. Mater. Med..

[14] Brett E., Flacco J., Blackshear C., Longaker M.T., Wan D.C. (2017). Biomimetics of Bone Implants: The Regenerative Road. Biores. Open Access.

[15] Patel K., Doyle C.S., Yonekura D., James B.J. (2010). Effect of Surface Roughness Parameters on Thermally Sprayed PEEK Coatings. Surf. Coat. Technol..

[16] Akkan C.K., Hammadeh M., Brück S., Park H.W., Veith M., Abdul-Khaliq H., Aktas C. (2013). Plasma and Short Pulse Laser Treatment of Medical Grade PEEK Surfaces for Controlled Wetting. Mater. Lett..

[17] Hussain S., Rutledge L., Acheson J.G., Meenan B.J., Boyd A.R. (2020). The Surface Characterisation of Polyetheretherketone (PEEK) Modified via the Direct Sputter Deposition of Calcium Phosphate Thin Films. Coatings.

[18] Ourahmoune R., Salvia M., Mathia T.G., Mesrati N. (2014). Surface Morphology and Wettability of Sandblasted PEEK and Its Composites: Surface Morphology and Wettability. Scanning.

[19] Wang S., Zhang S., Yang Y., Dong Z., Wang G. (2022). Direct Electrochemical Grafting of Crystalline PAEK Macromolecule on Carbon Fiber to Enhance the Interfacial Properties of PEEK/CF Composites. Compos. Sci. Technol..

[20] Bradford J.P., Hernandez-Moreno G., Pillai R.R., Hernandez-Nichols A.L., Thomas V. (2023). Low-Temperature Plasmas Improving Chemical and Cellular Properties of Poly (Ether Ether Ketone) Biomaterial for Biomineralization. Materials.

[21] Czwartos J., Budner B., Bartnik A., Wachulak P., Butruk-Raszeja B.A., Lech A., Ciach T., Fiedorowicz H. (2021). Effect of Extreme Ultraviolet (EUV) Radiation and EUV Induced, N2 and O2 Based Plasmas on a PEEK Surface’s Physico-Chemical Properties and MG63 Cell Adhesion. Int. J. Mol. Sci..

[22] Yakufu M., Wang Z., Wang Y., Jiao Z., Guo M., Liu J., Zhang P. (2020). Covalently Functionalized Poly(Etheretherketone) Implants with Osteogenic Growth Peptide (OGP) to Improve Osteogenesis Activity. RSC Adv..

[23] Noiset O., Schneider Y.-J., Marchand-Brynaert J. (1999). Fibronectin Adsorption or/and Covalent Grafting on Chemically Modified PEEK Film Surfaces. J. Biomater. Sci. Polym. Ed..

[24] Noiset O., Schneider Y.-J., Marchand-Brynaert J. (1997). Surface Modification of Poly(Aryl Ether Ether Ketone) (PEEK) Film by Covalent Coupling of Amines and Amino Acids through a Spacer Arm. J. Polym. Sci. A Polym. Chem..

[25] Cassari L., Zamuner A., Messina G.M.L., Marsotto M., Chen H., Gonnella G., Coward T., Battocchio C., Huang J., Iucci G. (2023). Bioactive PEEK: Surface Enrichment of Vitronectin-Derived Adhesive Peptides. Biomolecules.

[26] Cassari L., Zamuner A., Messina G.M.L., Marsotto M., Chang H., Coward T., Battocchio C., Iucci G., Marletta G., Di Silvio L. (2023). Strategies for the Covalent Anchoring of a BMP-2-Mimetic Peptide to PEEK Surface for Bone Tissue Engineering. Materials.

[27] Thumshirn G., Hersel U., Goodman S.L., Kessler H. (2003). Multimeric Cyclic RGD Peptides as Potential Tools for Tumor Targeting: Solid-Phase Peptide Synthesis and Chemoselective Oxime Ligation. Chem. Eur. J..

[28] Shao J., Tam J.P. (1995). Unprotected Peptides as Building Blocks for the Synthesis of Peptide Dendrimers with Oxime, Hydrazone, and Thiazolidine Linkages. J. Am. Chem. Soc..

[29] Dettin M., Zamuner A., Roso M., Iucci G., Samouillan V., Danesin R., Modesti M., Conconi M.T. (2015). Facile and Selective Covalent Grafting of an RGD-Peptide to Electrospun Scaffolds Improves HUVEC Adhesion. J. Pept. Sci..

[30] Secchi V., Franchi S., Ciccarelli D., Dettin M., Zamuner A., Serio A., Iucci G., Battocchio G. (2019). Biofunctionalization of TiO_2_ Surfaces with Self-Assembling Layers of Oligopeptides Covalently Grafted to Chitosan. ACS Biomater. Sci. Eng..

[31] Guan T., Li J., Chen C., Liu Y. (2022). Self-Assembling Peptide-Based Hydrogels for Wound Tissue Repair. Adv. Sci..

[32] Anselme K., Bigerelle M., Dufresne E., Judas D., Iost A., Hardouin P. (2000). Qualitative and quantitative study of human osteoblast adhesion on materials with various surface roughnesses. J. Biomed. Mat. Res..

[33] Becker M., Lorenz S., Strand D., Vahl C.-F., Gabriel M. (2013). Covalent Grafting of the RGD-Peptide onto Polyetheretherketone Surfaces via Schiff Base Formation. Sci. World J..

[34] Chelushkin P.S., Leko M.V., Dorosh M.Y., Burov S.V. (2017). Oxime ligation in acetic acid: Efficient synthesis of aminooxy-peptide conjugates. J. Pept. Sci..

[35] Liu F., Hakami R.M., Dyas B., Bahta M., Lountos G.T., Waugh D.S., Ulrich R.G., Burke T.R. (2010). A rapid oxime linker-based library approach to identification of bivalent inhibitors of the yersinia pestis protein-tyrosine phosphatase, yoph. Bioorg. Med. Chem. Lett..

[36] Bendale A.R., Bhatt R., Nagar A., Jadhav A.G., Vidyasagar G. (2011). Shiff base synthesis by unconventional route: An innovative green approach. Der Pharma Chem..

[37] Secchi V., Franchi S., Dettin M., Zamuner A., Beranová K., Vladescu A., Battocchio C., Graziani V., Tortora L., Iucci G. (2020). Hydroxyapatite surfaces functionalized with a self-assembling peptide: XPS, RAIRS and NEXAFS study. Nanomaterials.

[38] (2021). Geometrical Product Specifications (GPS)—Surface Texture: Area l, Part 2: Terms, Definitions, and Surface Texture Parameters.

[39] Brun P., Ghezzo F., Roso M., Danesin R., Palù G., Bagno A., Modesti M., Castagliuolo I., Dettin M. (2011). Electrospun scaffolds of self-assembling peptides with poly(ethylene oxide) for bone tissue engineering. Acta Biomater..

[40] Day T.F., Guo X., Garrett-Beal L., Yang Y. (2005). Wnt/β-Catenin Signaling in Mesenchymal Progenitors Controls Osteoblast and Chondrocyte Differentiation during Vertebrate Skeletogenesis. Dev. Cell.

[41] McKee M.D., Pedraza C.E., Kaartinen M.T. (2011). Osteopontin and wound healing in bone. Cells Tissues Organs.

[42] Chen Q., Shou P., Zhang L., Xu C., Zheng C., Han Y., Shi Y. (2014). An osteopontin-integrin interaction plays a critical role in directing adipogenesis and osteogenesis by mesenchymal stem cells. Stem Cells..

[43] Schvartz I., Seger D., Shaltiel S. (1999). Vitronectin. Int. J. Biochem. Cell Biol..

[44] Salasznyk R.M., Williams W.A., Boskey A., Batorsky A., Plopper G.E. (2004). Adhesion to vitronectin and collagen I promotes osteogenic differentiation of human mesenchymal stem cells. J. Biomed. Biotech..

[45] Bolotin D.S., Bokach N.A., Demakova M.Y., Kukushkin V.Y. (2017). Metal-Involving Synthesis and Reactions of Oximes. Chem. Rev..

[46] (2012). NIST X-Ray Photoelectron Spectroscopy Database, Version 4.1.

[47] Secchi V., Franchi S., Santi M., Dettin M., Zamuner A., Iucci G., Battocchio C. (2018). Self-Assembling Behavior of Cysteine-Modified Oligopeptides: An XPS and NEXAFS Study. J. Phys. Chem. C.

[48] Engler A.J., Richert L., Wong J.Y., Picart C., Discher D.E. (2004). Surface probe measurements of the elasticity of sectioned tissue, thin gels and polyelectrolyte multilayer films: Correlations between substrate stiffness and cell adhesion. Surf. Sci..

