# Supplementary material for: Polyetheretherketone Double Functionalization with Bioactive Peptides Improves Human Osteoblast Response
Source: Biomimetics (Basel). 2024 Dec 17;9(12):767. doi: 10.3390/biomimetics9120767 (PMC11673530; doi:10.3390/biomimetics9120767)
Supplement: Supplementary file 1 [file biomimetics-09-00767-s001.zip › biomimetics-3276698-supplementary.pdf]

Supplementary Materials

Polyetheretherketone Double Functionalization with Bioactive Peptides Improves Human Osteoblast Response

Leonardo Cassari [1], Cristian Balducci [1], Grazia M. L. Messina [2], Giovanna Iucci [3], Chiara Battocchio [3], Federica Bertelà [3], Giovanni Lucchetta [1], Trevor Coward [4], Lucy Di Silvio [4], Giovanni Marletta [2], Annj Zamuner [1,5], Paola Brun [6] and Monica Dettin [1,*]

[1] Department of Industrial Engineering, University of Padova, Via Marzolo 9, 35131 Padova, Italy
[2] Laboratory for Molecular Surface and Nanotechnology (LAMSUN), Department of Chemical Sciences, University of Catania and CSGI, Viale A. Doria, 6, 95125 Catania, Italy
[3] Department of Science, Roma Tre University, Via della Vasca Navale 79, 00146 Roma, Italy;
[4] Faculty of Dentistry, Oral & Craniofacial Sciences, King's College London, London SE1 9RT, UK;
[5] Department of Civil, Architectural and Environmental Engineering, University of Padova, Via Marzolo 9, 35131 Padova, Italy
[6] Department of Molecular Medicine, University of Padova, Via A. Gabelli 63, 35121 Padova, Italy
* Correspondence: monica.dettin@unipd.it; Tel.: +39-049-827-5553

Supplementary Materials

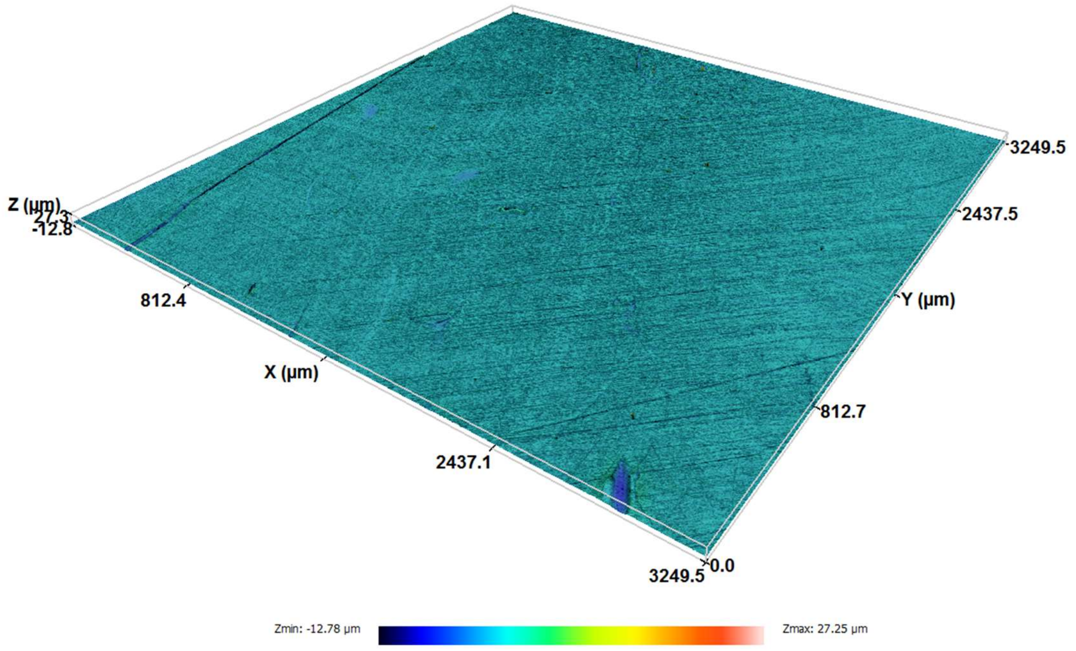

Figure S1. Profilometer microimages obtained on smooth PEEK

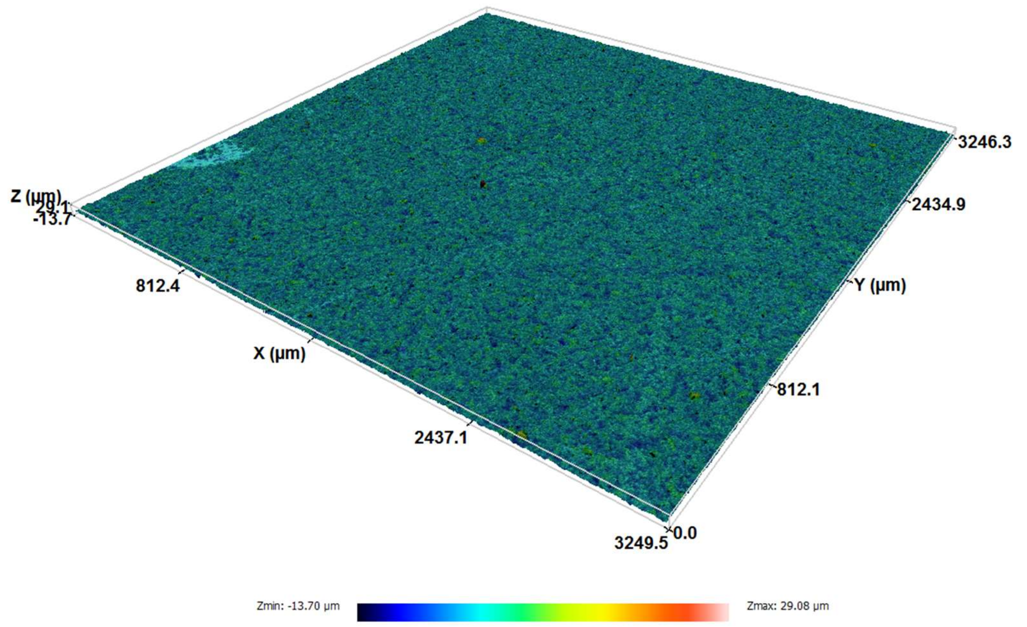

Figure S2. Profilometer microimages obtained on R60-PEEK

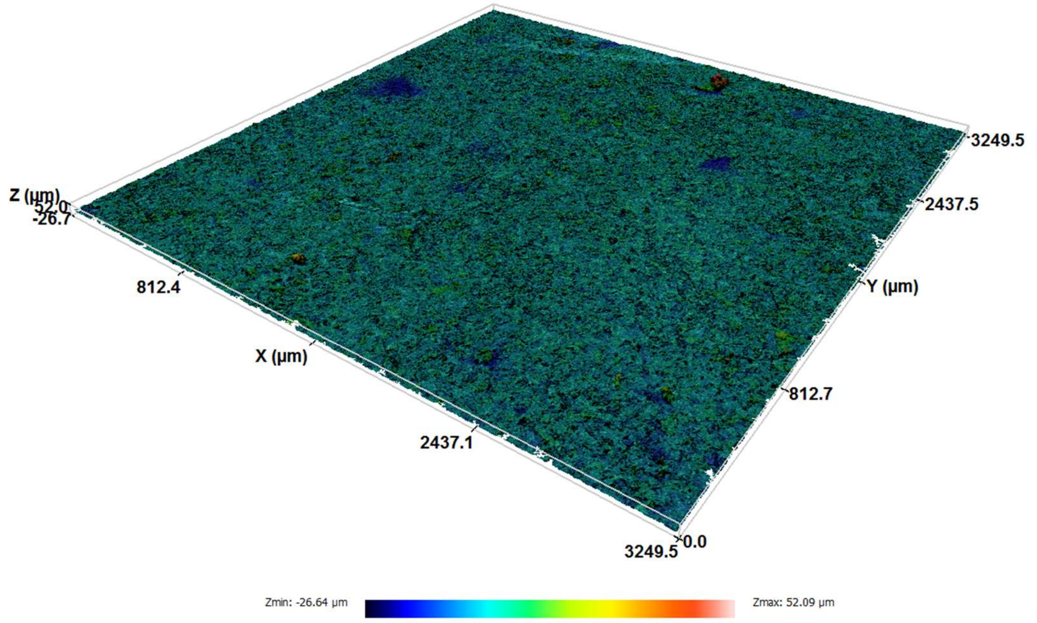

Figure S3. Profilometer microimages obtained on R110-PEEK

Table S1: XPS data (BE, FWHM, atomic ratios) of PEEK, PEEK+Aoa-EAK in 40 mM monobasic sodium phosphate at pH 6 and PEEK+Aoa-EAK in 1eq oh AcOH in DMSO samples.

Sample	Signal	Assignment	BE (eV)	FWHM	Internal Atomic ratios (%)	Atomic ratios (%)
**PEEK**	C1s	C-C	284.7	1.52	73	61
		C-O	286.3		23	19
		C=O	287.7		4	3
	O1s	C=O	531.4	1.79	51	8
		C-O	533.4		49	8
**PEEK+Aoa-EAK in 40 mM monobasic sodium phosphate at pH 6**	C1s	C-C	284.7	1.59	67	50
		C-N, C-O	286.2		23	17
		C=O	287.3		5	4
		N-C=O	288.3		5	4
	N1s	C-N	299.9	1.57	83	3
		$-N^{+}$	401.2		17	0.6
	O1s	C=O	531.7	2.04	65	14
		C-O	533.3		35	8
**PEEK+Aoa-EAK in 1eq of AcOH in DMSO**	C1s	C_{ar}, C-C	284.7	1.59	62.6	45.3
		C-N, C-O	286.2		23.6	17.1
		C=O	287.4		6.8	4.9
		N-C=O	288.5		4	2.9
	N1s	C-N	399.9	1.9	90.7	4.9
		$-N^{+}$	401.0		9.3	0.5
	O1s	C=O	531.5	1.9	49	10.9
		C-O	533.2		51	11.3
